# Tunable Graphene-based Plasmonic Perfect Metamaterial Absorber in the THz Region

**DOI:** 10.3390/mi10030194

**Published:** 2019-03-18

**Authors:** Zao Yi, Jiajia Chen, Chunlian Cen, Xifang Chen, Zigang Zhou, Yongjian Tang, Xin Ye, Shuyuan Xiao, Wei Luo, Pinghui Wu

**Affiliations:** 1 School of Science, Southwest University of Science and Technology, Mianyang 621010, China; yizaomy@swust.edu.cn (Z.Y.); chenjiajia522@outlook.com (J.C.); cenchunlian@mails.swust.edu.cn (C.C.); chenxifang1988@yeah.net (X.C.); zhouzigang@swust.edu.cn (Z.Z.); tangyongjian2000@sina.com (Y.T.); 2 Research Center of Laser Fusion, China Academy of Engineering Physics, Mianyang 621900, China; yexin@caep.cn; 3 Institute for Advanced Study, Nanchang University, Nanchang 330031, China; syxiao@ncu.edu.cn; 4 Central South University, Changsha 410013, China; dkny2013@outlook.com; 5 Photonic Technology Research & Development Center, Key Laboratory of Information Functional Material for Fujian Higher Education, Quanzhou Normal University, Fujian 362000, China

**Keywords:** perfect metamaterial absorber, FDTD method, graphene, surface plasmon resonance

## Abstract

The optical performance of a periodically tunable plasma perfect metamaterial absorber based on a square-square-circle array we propose in the terahertz region is analyzed in this work by the finite difference time domain (FDTD) method. We not only discuss the impact of various parameters such as period *a*, length *L*, radius *R*, and incident angle *θ* under transverse magnetic (TM)- and transverse electric (TE)-polarization on the absorption spectra of the absorber but also study the effect of the Fermi energy *E_F_* and relaxation time *τ*. Finally, we simulate the spectra as the surrounding refractive index *n* changes to better evaluate the sensing performance of the structure, producing a sensitivity *S* of the structure of up to 15006 nm/RIU. On account of this research, we find that the absorber is beneficial to sensors and detectors in the terahertz region.

## 1. Introduction

Whether through the development of terahertz technology [1,2,3,4] or the development of perfect absorbers [5,6,7], the emergence of metamaterials has undoubtedly played a role in promoting them. Although terahertz technology has made rapid progress in light sources and detectors [8,9,10,11], it still needs further exploration and research compared with the mature infrared and microwave bands. However, if we want to continue to develop terahertz (THz) technology, we need to solve a difficult problem: finding a kind of material that can strongly respond to the terahertz band. Metamaterials are the best choice available to solve this problem [12]. Because the high absorption of light is of great significance to optical detectors and photovoltaic power generation, the high absorptivity of light, even perfect absorption, has attracted great attention. Perfect absorption plays a major role in the application of devices such as filters, modulators, switches, photodetectors, sensors, solar cells, and so on [13,14,15,16,17,18]. Metamaterials have become one of the most promising materials in attempts to achieve perfect absorption [19]. The response of metamaterials to light is not determined by their composition but by their structure. Hence, the designed metamaterials often have some electromagnetic properties that natural materials do not possess, such as having a perfect lens and negative refractive index [20,21]. In a word, the design of metamaterial absorbers with strong responses in the terahertz band has become a hot research topic. 

Graphene has attracted much interest because of its high carrier mobility and the advantages of doping or having regular structure patterning in the broadband region [22,23]. Graphene supports surface plasmon resonance (SPR) in the infrared and terahertz bands [24,25,26,27,28]. Thus, when graphene binds to metamaterials, metamaterials can enhance light absorption by exciting surface plasmon resonance [29]. 

In our work, we propose a periodically tunable plasma perfect metamaterial absorber based on a square-square-circle array in the terahertz region. With silicon as the base, gold, silicon dioxide, and graphene are added layer by layer in order. Because of the flexibility of the structure proposed above, we can adjust its response to light by changing the structural parameters. Across the whole research process, we discuss the impact of not only structural parameters, but also Fermi energy, relaxation time, and the angle of incidence under different polarizations on absorption. Lastly, we also explore the sensing performance of this structure. Hence, the structure we propose can be available for sensors and detectors in the terahertz region.

## 2. Design of Structure and Research Method

**Figure 1:**
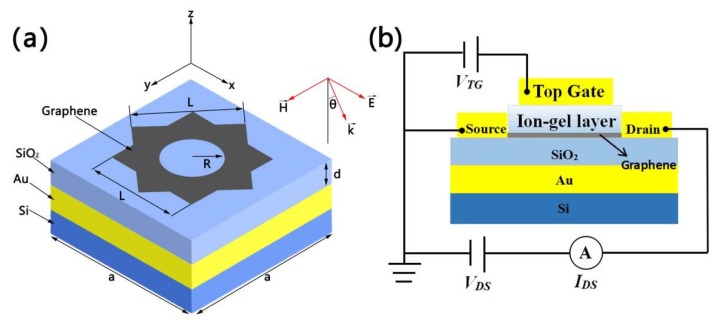
(**a**) Diagrammatic sketch of a unit cell of the square-square-circle graphene array with its geometric parameters and the polarization and propagation of the light source. (**b**) Principle geometry of the top grid structure for the operating Fermi energy of graphene. V_TG_, V_DS_ and I_DS_ express the top gate voltage, the voltage and current flowing between Source and Drain, respectively.

A diagrammatic sketch of our square-square-circle graphene array is shown in Figure 1a. With silicon as the base, gold, silicon dioxide, and graphene were added layer by layer in order. Here, we set the refractive index of Si and the relative permittivity of SiO_2_ to 3.4 and 3.9 [30,31], respectively. The shape of graphene consists of two overlapping squares with length *L* and a circle with radius *R* which is cut out of the center of the square. The thickness of the whole graphene layer *t_g_* is 1 nm. The other parameters *a*, *d*, and *θ* represent the period of the array, the thickness of SiO_2_, and angle of incident light, respectively. 

It is known because of Equation (1) that we can adjust the surface conductivity of graphene by changing the Fermi energy. In the mid-infrared and terahertz bands, gate dielectrics such as indium-tinoxide [32,33], monolayer MoS_2_ [34], 2D electron gas [35,36], and ion-gel [37,38] have been explored in large quantities. At present, the Fermi energy of graphene can be increased by low top gate voltage. The most effective dielectric is an ion-gel with high capacitance. Because of its good mechanical flexibility, fatigue stability, and excellent electrochemical and thermal stability, it can be compatible with tunable graphene plasmonic devices on various substrates [39]. The structure diagram of the ion-gel top gate which can be used to control the Fermi energy of graphene is shown in Figure 1b. The ion-gel layer, which induces carrier concentration and allows the absorber to enter the terahertz band [40,41,42], is inserted between the graphene and gold electrodes.

The Kubo formula, which is made up of interband and intraband transitions, can describe the surface conductivity of graphene *σ* [30,43]. In the terahertz region, the intraband transition plays a major role as *E_F_* >> *K_B_T*, where *K_B_* and *T* are the Boltzmann constant and temperature, respectively [44]. Hence, the surface conductivity of graphene *σ* which is similar to the Drude-like model [45,46,47,48] can be expressed as

(1) $$\sigma (\omega )=\frac{ie^{2}E_{F}}{\pi \hbar^{2}(\omega +i/\tau )}$$

where *e*, *ħ*, *ω*, *E_F_,* and *τ* are the electron charge, reduced Plank’s constant, frequency of incident light, Fermi energy, and relaxation time, respectively [49]. In addition, the permittivity of graphene is isotropic in the plane of the thin plate and non-dispersive out of the plane, which can be described in diagonal tensor form using [50,51,52]

(2) $$\varepsilon_{xx}=\varepsilon_{yy}=2.5+\frac{i\sigma (\omega )}{\varepsilon_{0}\omega t_{g}}$$

(3) $$\varepsilon_{zz}=2.5$$

where *ε*_0_ is the permittivity of a vacuum.

In the simulation, we used the finite-difference time-domain (FDTD Solutions, Lumerical Inc., Vancouver, BC, Canada) method to obtain the data. We set periodic boundary conditions in the *x* and *y* directions, respectively. In the *z* direction of the incident plane wave propagation, perfectly matched layers were utilized. Then, the absorption can be expressed as follows: *A*(*ω*) = 1 − *R*(*ω*) − *T*(*ω*), where *R*(*ω*) = *|S*11(*ω*)*|*^2^ and *T*(*ω*) = *|S*21(*ω*)*|*^2^, gained by *S*-parameters, are reflection and transmission, respectively. Therefore, the perfect absorption can be discerned by reducing reflection and transmission to zero. In our structure, we set the thickness of the gold layer to 550 nm, which is larger than the skin depth of electromagnetic waves [53]. Hence, transmission can be effectively eliminated.

## 3. Simulation Results and Discussions

**Figure 2:**
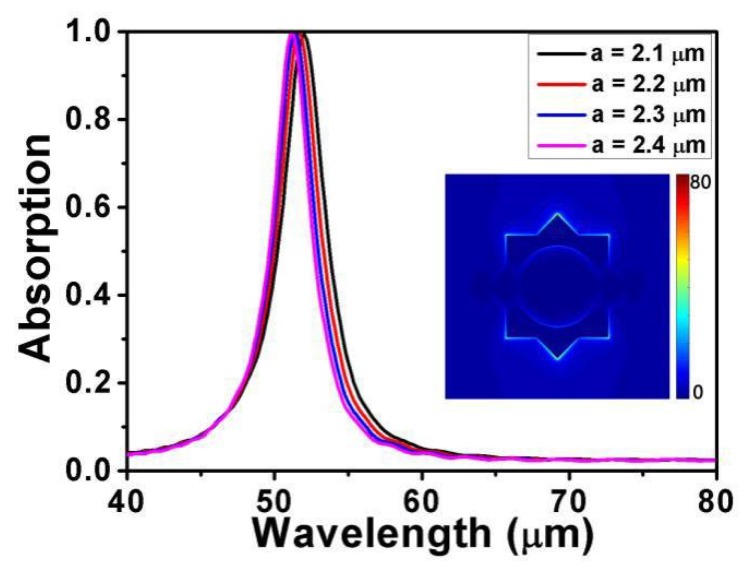
Absorption spectra for different values of period *a*. Other parameters remain fixed. The inserts indicate the distributions of the electric field (|E|) at *a* = 2200 nm.

At *L* = 1000 nm, *d* = 4200 nm, *R* = 400 nm, *θ* = 0°, *E_F_* = 0.9 eV, and *τ* = 0.9 ps, the simulated absorption spectra of the absorber under different periods are plotted in Figure 2. Here, we can see that the resonance wavelength has a blue shift and the absorption peak is almost unchanged with the incremental period. In other words, the period has a great influence on the resonance wavelength but less on the absorption peak. The symmetry of the perfect metamaterial absorber design keeps the absorption almost unchanged with various periods [54]. The inserts indicate the distributions of the electric field (|E|) at *a* = 2200 nm. It can be clearly seen that the electric field is localized at both ends of the structure, which is caused by the strong electric dipole resonance. This resonance can effectively catch the energy of light and produces enough time to eliminate the ohm loss in graphene. In addition, the reflection of gold reduces transmission to zero, which promotes perfect absorption.

**Figure 3:**
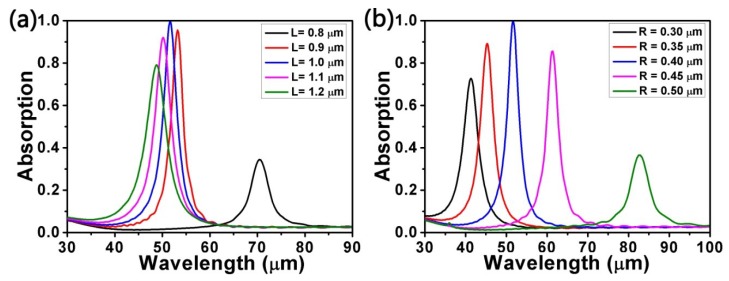
Absorption spectra for (**a**) different lengths *L* of the square and (**b**) different radii *R* of the circle. Other parameters remain fixed.

Here, we analyze the influence of different lengths *L* of the square on the absorption with *a* = 2200 nm, *d* = 4200 nm, *R* = 400 nm, *θ* = 0°, *E_F_* = 0.9 eV, and *τ* = 0.9 ps. As shown in Figure 3a, as the length *L* of the square rises from 0.8 μm to 1.2 μm, the resonance wavelength moves to a shorter wavelength, that is, a blue shift occurs. However, the value of the absorption peak experiences a different situation: rising and falling can be observed for *L* < 1.0 μm and *L* > 1.0 μm, respectively. That is to say, when *L* = 1.0 μm, the absorption is nearly perfect. As a result of the impedance matching condition [19] of the structure which is partially up to the parameter *L*, we can change the absorption peak. Hence, we observe a higher impedance matching condition as *L* rises. Then, an almost perfect impedance matching condition is gained when *L* = 1.0 μm. Nevertheless, if we continue to increase *L*, the impedance will mismatch.

To learn more about the influence of structural parameters on the absorption, we also considered how different radii *R* of the circle impacted the absorption with *L* = 1000 nm, *d* = 4200 nm, *a* = 2200 nm, *θ* = 0°, *E_F_* = 0.9 eV, and *τ* = 0.9 ps, as seen in Figure 3b. It is obvious that when *R* is raised from 0.30 μm to 0.50 μm at intervals of 0.05 μm, the resonance wavelength undergoes a red shift and the absorption goes through a process of increasing initially and then decreasing. Owing to the almost perfect impedance matching condition, the absorption peak reaches a maximum. The above simulation results also illustrate the fact that we can modulate the spectral responses in a way that optimizes the structural parameters *L* and *R*.

**Figure 4:**
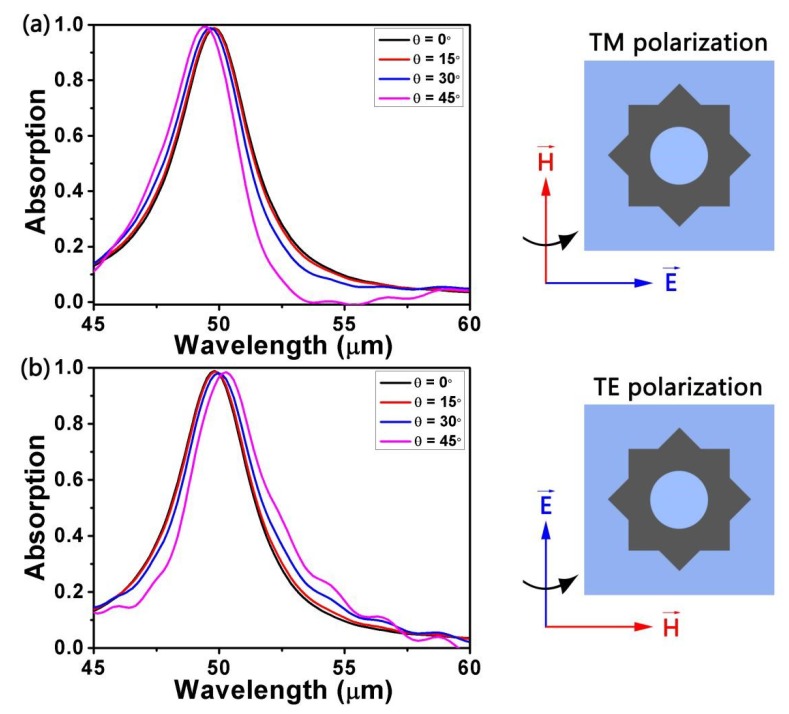
Absorption spectra at different incident angles for (**a**) transverse magnetic (TM)-polarization and (**b**) transverse electric (TE)-polarization.

Next, we discuss the impact of different incident angles *θ* on absorption under TM-polarization and TE-polarization, respectively. TM- and TE-polarization represent the direction of the incidence electric field along the x and y axes, respectively. The other parameters were set as *L* = 1000 nm, *d* = 4200 nm, *R* = 400 nm, *a* = 2200 nm, *E_F_* = 0.9 eV, and *τ* = 0.9 ps. From Figure 4, it can clearly be seen that in any polarization state, the absorption rates slightly increase or decrease with the incremental incident angle *θ* because of the varying reflectivity. However, in general, they remain above 0.95. The difference between TM polarization and TE polarization is that the resonance wavelength of the former is blue-shifted and the latter is red-shifted.

**Figure 5:**
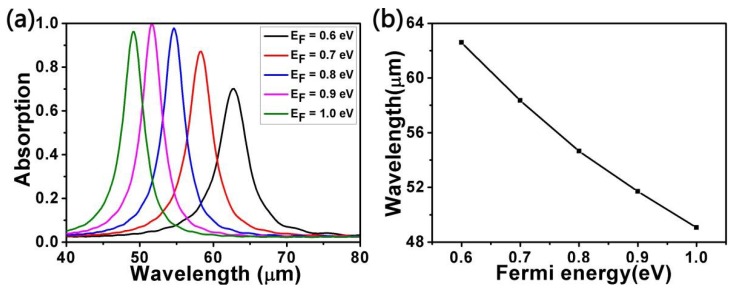
(**a**) Absorption spectra for different Fermi energy (*E_F_*) values with other parameters fixed. (**b**) The relationship between Fermi energy and the resonance wavelength.

As is known, Fermi energy *E_F_*, which can be regulated by applying a gate voltage and by means of chemical doping, has a deep influence on the surface conductivity of graphene. The connection between Fermi energy *E_F_* and the gate voltage *V* can be expressed [55] as *E_F_ = ħV_f_ (πε_0_ε_r_V/(et_s_))*, where *V_f_*, *ε_0_*, *ε_r_,* and *t_s_* are the Fermi velocity of graphene, the permittivity of a vacuum, the permittivity of the ion-gel and the thickness of the ion-gel, respectively. Therefore, we evaluated the effect of different Fermi energy values on absorption, as seen in Figure 5a. The other parameters were set as *L* = 1000 nm, *d* = 4200 nm, *R* = 400 nm, *a* = 2200 nm, *θ* = 0°, and *τ* = 0.9 ps. We can clearly see that the resonance wavelength experiences a blue shift and the wavelength peak first increases and then decreases with incremental Fermi energy. In Figure 5b we give the functional relationship between Fermi energy and the resonance wavelength. Obviously, there is almost a linear relationship between them. In order to better comprehend this, we propose a physical mechanism involving the charge carrier rising in number with incremental Fermi energy, which contributes to a stronger surface plasmon resonance (SPR) excitation [56,57]. The SPR reaches a maximum as the value of the Fermi energy is 0.9 eV. However, if the Fermi energy continues to increase on the basis of 0.9 eV, the resonance wavelength will depart from the strong SPR region. This is the reason why the wavelength peak increases first and then decreases with incremental Fermi energy.

**Figure 6:**
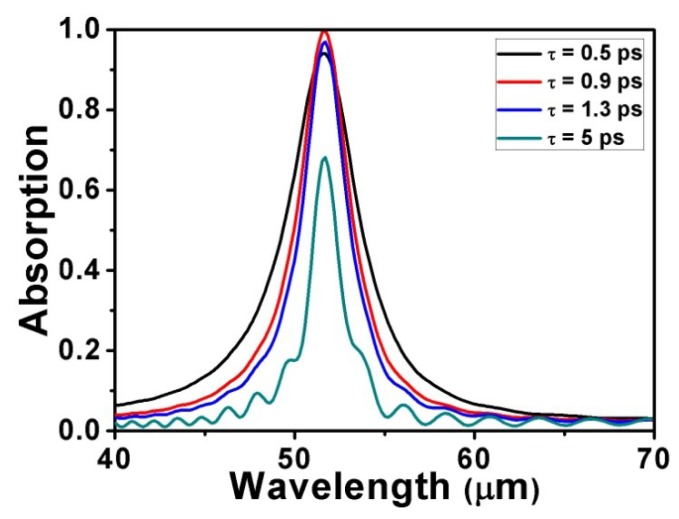
Absorption spectra for different relaxation times with other parameters fixed.

The formula *τ = E_F_·μ/(eV_F_^2^)*, with the Fermi energy *E_F_* = 0.9 eV, the Fermi velocity *V_F_* = 10^6^ m/s, and the carrier mobility *μ* = 1 m^2^/Vs, can be used to describe the relaxation time for graphene. The carrier mobility available can be enhanced by the placement of organic molecules on graphene, thus increasing the relaxation time [58]. As shown in Figure 6, the spectra show changes in absorption at different relaxation times with *L* = 1000 nm, *d* = 4200 nm, *R* = 400 nm, *a* = 2200 nm, *E_F_* = 0.9 eV, and *θ* = 0°. It is significant that the absorption peak rises when the relaxation time increases from 0.4 eV to 1 eV, while the absorption peak declines when the relaxation time increases from 1 eV to 1.2 eV. There is no doubt that when the relaxation time is equal to 1 eV, the peak is at its maximum. The reason for this phenomenon is that the charge carriers, which are conducive to plasma oscillation absorption, reach the saturation state when the relaxation time is equal to 1 eV [56,59]. If the relaxation time keeps increasing, most energy will be reflected, and the absorption will decrease. Hence, the absorption is no longer nearly perfect.

**Figure 7:**
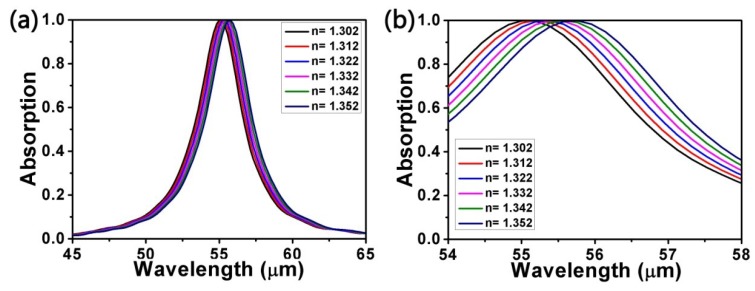
(**a**) Absorption spectra for different values of surrounding refractive index *n*. Other parameters are unchanged. (**b**) Local details of absorption peaks.

In order to better evaluate the sensing performance of the structure, we simulated the spectra as the surrounding refractive index *n* changes. The other parameters were set as *L* = 1000 nm, *d* = 4200 nm, *R* = 400 nm, *a* = 2200 nm, *θ* = 0°, *E_F_* = 0.9 eV, and *τ* = 0.9 ps. As shown in Figure 7, it is clear that when *n* is increased from 1.302 to 1.352 at intervals of 0.01, the absorption peak remains almost unchanged, but the resonance wavelength experiences a red shift. In other words, the surrounding refractive index *n* has a significant effect on the resonance wavelength but little on the absorption peak. This also indicates the fact that the structure is sensitive to changes in the surrounding refractive index. Furthermore, the absorption peak remains nearly always perfect. Hence, we can say that the structure can be applied to the sensors.

**Figure 8:**
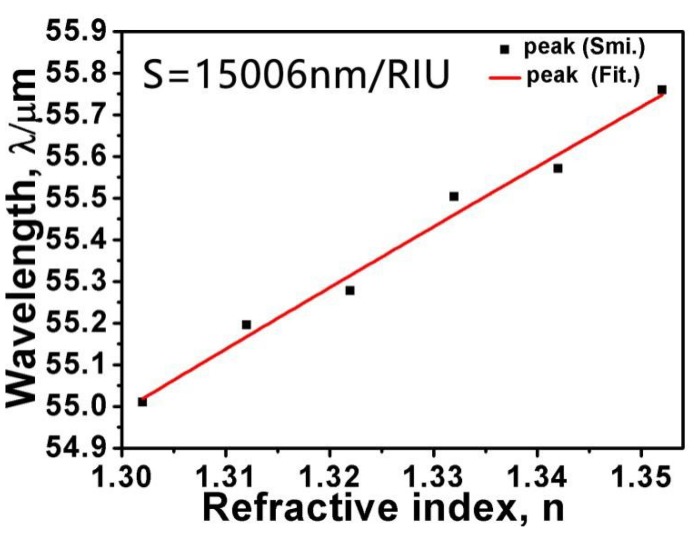
Simulated resonance wavelengths (black spots) and linear fit (red line) as a function of different values of surrounding refractive index *n*.

As is known, the sensitivity *S* and the figure of merit *FOM* can be used to define the sensing property [60]. The sensitivity can be stipulated as *S* = *∆λ/∆n* and the figure of merit can be expressed as *FOM* = *S/FWHM*. Here, *∆λ* and *∆n* are the change values of resonance wavelength and surrounding refractive index. *FWHM* is the full width at half maximum of absorption. In Figure 8, we illustrate the relationship between the surrounding refractive index *n* and the resonance wavelength. Each black dot corresponds to simulated data and the red line is the linear fit. The slope of the red line is expressed as the value of sensitivity *S*. As Figure 8 shows, the sensitivity *S* is approximately 15,006 nm/RIU. According to our formula, we obtain *FOM =* 4.19. Using previously published works on absorbers [59,60,61,62,63,64,65,66], Table 1 provides a comparison of their sensitivity. Compared with these sensors, we can see that the proposed structure has better sensing performance. Therefore, the structure we have proposed can be used as a sensor detector to detect changes in the surrounding environment.

## 4. Discussion

In this study, we analyzed the optical performance of a periodical tunable plasmonic perfect metamaterial absorber based on a square-square-circle graphene array that we proposed in the terahertz region, by FDTD. Simulated results revealed that we can change the structural parameters to tune the absorption peak and the resonance wavelength of the absorber due to the impedance matching condition. Furthermore, on account of the surface plasmon resonance, the spectra of the absorber can be adjusted by the Fermi energy *E_F_*. Additionally, relaxation time may only be used for controlling the absorption intensity because of the little effect on the resonance wavelength. Finally, simulation of the spectra as the surrounding refractive index *n* changes can produce a sensitivity *S* of the structure of up to 15006 nm/RIU, which means that it is beneficial for a sensor detector to detect changes in the surrounding environment. Consequently, the absorber we proposed is beneficial to sensors and detectors in the terahertz region.

## Figures and Tables

**Table 1:** Sensitivity performance comparison between different absorber designs proposed in previous publications.

Reference	[60]	[61]	[62]	[63]	[64]	[65]	[66]	Proposed
Sensitivity (μm/RIU)	9.59	0.43	1.015	0.35	1.445	2.4	0.885	15.006
